# Overview and Application of Intrasubject Variability in ADRD Research

**DOI:** 10.1002/alz.084210

**Published:** 2025-01-03

**Authors:** Michael H. Malek‐Ahmadi

**Affiliations:** ^1^ Banner Alzheimer’s Institute, Phoenix, AZ USA; ^2^ Arizona Alzheimer’s Consortium, Phoenix, AZ USA; ^3^ University of Arizona College of Medicine‐Phoenix, Phoenix, AZ USA

## Abstract

Intrasubject variability is an important, but often overlooked, measure that has shown to be predictive of important clinical outcomes in ADRD research. Intrasubject variability is often quantified using the intrasubject standard deviation (ISD) that is derived from longitudinal measures or cross‐sectional observations from similarly scaled variables. This talk will begin with an overview of ISD and its application in both longitudinal and cross‐sectional analyses. For longitudinal analyses, ISD can be an important measure of normative performance variability on cognitive measures which can better inform clinicians on what normative trajectories for cognitive tests might be. An example using longitudinal data for the Montreal Cognitive Assessment (MoCA) will demonstrate how the ISD can be used quantify normative variability of longitudinal cognitive performance. The second part of this talk will discuss how ISD is used in cross‐sectional analyses of neuropsychological data. Specifically, how ISD is used to characterize the concept of dispersion which quantifies the inconsistency of between‐domain cognitive performance. Several studies have indicated that cognitive dispersion predicts incident cognitive decline and is associated with AD pathology which highlight the utility ISD may have in characterizing preclinical AD. An additional example using data from a complex motor task will show how the ISD of repeated task trials can differentiate cognitively unimpaired (CU), mild cognitive impairment (MCI) and AD cases. The conceptualization and application of ISD in this talk will set the stage for the other talks in this session that will demonstrate in greater detail how ISD can be used across the AD spectrum.

